# Preparation of Low Molecular Weight Liquid Polybutadiene Rubber Featuring High 1,4 Content by Nickel-Based Ziegler–Natta Catalytic System

**DOI:** 10.3390/polym18091051

**Published:** 2026-04-26

**Authors:** Hongfei Sun, Heng Liu, Xuequan Zhang, Feng Wang

**Affiliations:** 1 State Key Laboratory of Advanced Optical Polymer and Manufacturing Technology, Qingdao 266042, China; s13345013602@163.com (H.S.); xqzhang@qust.edu.cn (X.Z.); 2 Key Laboratory of Rubber-Plastics, Ministry of Education, Qingdao 266042, China; 3 School of Polymer Science and Engineering, Qingdao University of Science & Technology, Qingdao 266042, China

**Keywords:** nickel, low molecular weight, high *cis*-1,4, polybutadiene

## Abstract

A ligand-free Ni(acac)_2_/EASC Ziegler–Natta catalytic system was developed for the efficient synthesis of low molecular weight liquid polybutadiene (LPB) featuring high 1,4 content. The influences of key polymerization parameters, including Al/Ni ratio, polymerization temperature, monomer-to-catalyst ratio ([Bd]/[Ni]), and external donors, were systematically investigated to elucidate structure–reactivity relationships. Increasing the Al/Ni ratio significantly enhances catalytic activity while promoting chain transfer reactions, leading to reduced molecular weights and broader molecular weight distributions, with minimal impact on overall 1,4 selectivity. Polymerization temperature strongly affects both activity and stereoselectivity; elevated temperatures accelerate chain transfer processes and broaden dispersity, while inducing a shift from kinetically favored *cis*-1,4 insertion toward increased *trans*-1,4 incorporation. Variation of the [Bd]/[Ni] ratio provides an effective handle for molecular weight regulation, where higher ratios favor chain propagation over chain transfer, affording higher molecular weights but lower monomer conversion. Notably, the system maintains consistently high 1,4 content (>98%) across a wide range of conditions. In contrast, the introduction of external donors markedly affects catalytic behavior depending on their coordination ability. Strongly coordinating O- and S-containing donors partially deactivate the catalyst and significantly shift regioselectivity toward 1,2-vinyl incorporation (up to ~20%), while N- and P-containing donors are well tolerated and can increase molecular weight by suppressing chain transfer pathways, which also results in products with higher 1,2 content.

## 1. Introduction

Liquid rubber represents a class of low molecular weight elastomeric materials that combine flowability with intrinsic rubber elasticity, offering valuable formulation advantages across a wide range of applications. In particular, liquid polybutadiene (LPB) is widely used as a binder or reactive intermediate in sealants, adhesives, castable elastomers, and energetic materials due to its low viscosity, excellent flexibility, and high chemical compatibility [1,2,3,4,5,6]. Beyond molecular weight considerations, the microstructure of LPB also plays a decisive role in determining its physical properties [7]. In particular, PB enriched in 1,4 units retains a flexible unsaturated backbone and exhibits very low glass transition temperatures, which translate into superior elasticity and outstanding low-temperature performance compared with 1,2-vinyl-rich analogs. For instance, Guo et al. demonstrated that cis-1,4-enriched LPB exhibits a remarkably low glass transition temperature (Tg) of −101 °C, which is significantly lower than that of polybutadienes with other microstructures. In addition, improved mechanical properties, including higher tensile strength (1.89 MPa) and elongation at break (up to 1100%), were observed, outperforming counterparts with higher vinyl content [8]. Based on these considerations, efficient approaches to accessing low molecular weight PB with high 1,4 content remain of significant academic interest and technological relevance.

**Scheme 1:**
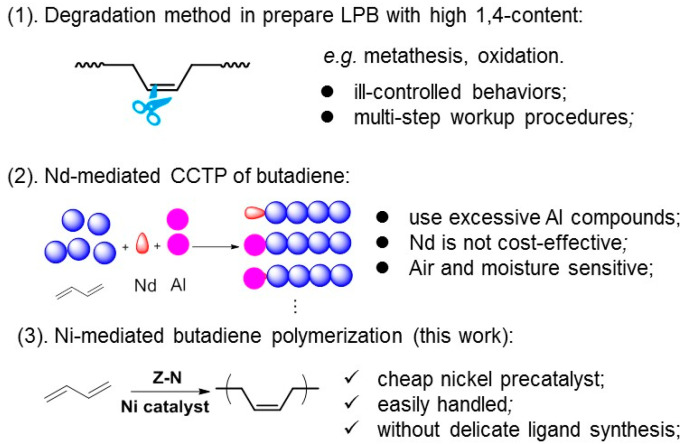
Procedures for preparing LPB with high 1,4 content.

Over the past decades, multiple strategies have been developed to synthesize LPB, including radical polymerization, anionic polymerization, coordination polymerization, ring-opening metathesis, and chemical degradation. Free-radical and anionic polymerization is widely practiced industrially for its robustness and tolerance to impurities, but typically yields polymers with high 1,2-vinyl microstructure. Independently, post-processing routes—including thermal or oxidative degradation and catalytic metathetic depolymerization—can convert high molecular weight PB into flowable materials (Scheme 1(1)) [9,10,11,12,13,14,15,16,17,18]. Nevertheless, these processes generally are accompanied by limited control over depolymerization behaviors, thus resulting in broad molecular weight distributions. In contrast, stereoregular coordination polymerization provides a more precise and modular route for microstructure regulation. In particular, rare earth metal systems, such as Nd-based catalysts, are well known for enabling exceptionally high *cis*-1,4 selectivity and highly controlled polymerization kinetics via coordinative chain transfer polymerization strategy (CCTP) (Scheme 1(2)), allowing predictable molecular weight growth and narrow dispersities [19,20,21,22,23,24,25,26,27,28,29,30,31,32]. However, despite these advantages, Nd-based methodologies face practical barriers that limit their widespread adoption for liquid rubber synthesis. The precatalysts are intrinsically expensive, and the coordinative chain transfer processes required to achieve low molecular weights depend on large amounts of organoaluminum chain transfer agents, further inflating costs and compromising atom economy. These considerations highlight the urgent need for alternative catalytic strategies that provide 1,4-enriched liquid PB while minimizing catalyst consumption and overall process cost.

Due to the inherent earth abundance, good tolerance to heteroatoms, easy handling, etc., late transition metal-mediated diene polymerization is currently widely used to access commercially available polydiene synthetic rubbers [33,34,35,36,37,38,39,40,41,42,43,44,45,46,47,48,49,50,51,52,53,54,55]. One of the most representative systems is the Ni(OCOR)_2_/BF_3_·OEt_2_/AlEt_3_ catalytic system, which is able to produce high molecular weight polybutadiene products with the assistance of the Lewis acid BF_3_·OEt_2_. In previous reports, it has been found that in the absence of such a strong Lewis acid, extensive chain transfer to the monomer can occur, which therefore enables the generation of low molecular weight liquid polybutadiene (LPB) [56,57,58,59,60]. During the past two decades, various nickel-based catalytic systems containing diversified ligands, such as *α*-diimines [40,61,62], pyridine-imines [43,63,64,65,66], phenanthroline [67], and so on, have been developed for butadiene polymerization [68]. These systems aim to enhance the overall catalytic activity, improve the *cis*-1,4 selectivity, or establish the structure/reactivity relationship. In contrast to these systems, we are seeking ligand-free nickel catalytic systems that can be directly used for diene polymerization to access LPB, because they are readily available for large-scale production without the tedious synthesis of ligands. It is of note that some ligand-free nickel catalysts have been disclosed in previous reports. For instance, in 1999, Endo reported the NiCl_2_/methylaluminoxane (MAO) system for the *cis*-1,4-selective polymerization of Bd to afford LPB with molecular weights (*M*_n_s) of around 1.2 × 10^4^ g/mol. In 1992, Sato reported a nickel naphthenate-based system (Ni(naph)_2_/AlEt_3_) for the preparation of *cis*-1,4-enriched LPB in the presence of external halogenated compounds, without which the Ni(naph)_2_/AlEt_3_ system would be completely inactive. In 1983, Dumas disclosed a nickel stearate-based system (Ni(stearate)_2_/Et_2_AlCl) for Bd polymerization and primarily investigated the influence of water on polymerization behaviors. Despite these advances, with the goal of low-cost, large-scale production of *cis*-1,4 LPB, these systems still have some shortcomings, including (1) the utilization of expensive MAO as a cocatalyst, generally, a higher amount of MAO is required to achieve acceptable catalytic activities; (2) the use of toluene as a solvent, which is not conducive to low-cost, large-scale production due to its higher boiling point and toxicity compared with alkanes, which are more commonly used in polybutadiene production in China; (3) the lack of an effective approach to regulate the ratio of *cis-*1,4 units-to-1,2 units. Based on this consideration, we herein report a Ni(acac)_2_-based Ziegler–Natta catalytic system Ni(acac)_2_/EASC for 1,3-butadiene polymerization to prepare LPB. The influences of polymerization conditions, such as the Al/Ni ratio, temperature, and external donors, were systematically investigated. Additionally, it is noteworthy that, distinct from the previous literature, which often uses aromatic solvents as the medium during polymerization to increase the solubility of the nickel precatalyst, we used hexane as the solvent throughout the investigation. This allows us to easily carry out the polymerization in a scale-up reactor without concerns about solvent recovery. Moreover, compared with the Nd-based CCTP systems, the present system employs much cheaper EASC as the cocatalyst, which requires a much smaller amount to achieve high catalytic activity, thus making it more economically favorable. Additionally, the introduction of external donors offers an effective strategy to regulate the microstructure of LPB, enabling precise control over *cis*-1,4 content ranging from high to medium and low levels.

## 2. Materials and Methods

### 2.1. Materials

Nickel naphthenate Ni(naph)_2_ was purchased from the Macklin Chemical Company (Shanghai, China) and used as a hexane solution (0.02 mol L^−1^). Al_2_Et_3_Cl_3_ (EASC) is a commercial product of Energy Chemical (Shanghai, China) and was diluted with hexane to 2.0 mol L^−1^. 1,3-Butadiene was supplied by Jinzhou Petrochemical Corporation (Jinzhou, China) and purified by passing through four columns packed with 4 Å and KOH prior to use. Hexane was refluxed over CaH_2_ under nitrogen and then distilled before use. Chemicals, such as 1,4-cyclohexanedione(CHD), 2-aminothiophenol (ATP), pentafluorophenol (PFP), dimestylboron fluoride (DMBF), 2-fluoro-6-methylaniline (FMA), azobisisobutyronitrile (AIBN), 2,2′-azobis(2,4-dimethylvaleronitrile) (AVBN), diethyl phosphite (DEP), and triphenyl phosphate (TPP), were all purchased from Energy Chemical (Shanghai, China) and used as received.

### 2.2. Analytical Method

The number average molecular weights (*M*_n_) and molecular weight distribution (*M*_w_/*M*_n_) of polymers were measured at 40 °C by using gel permeation chromatography (GPC) (Santa Clara, CA, USA) equipped with a Waters 515 HPLC pump, four columns (HMW 7 THF, HMW 6E THF × 2, HMW 2 THF), and a Waters 2414 refractive index detector. Tetrahydrofuran (THF) was used as eluent at a flow rate of 1.0 mL/min. Sample solutions were filtered through a 0.45 μm microfilter before injection. The relative values of *M*_n_ and *M*_w_/*M*_n_ were calculated by using polystyrene calibration. The column was calibrated with monodisperse polystyrene (PS) standards having the Mark–Houwink constants K = 2.46 × 10^−4^, α = 0.732 for polybutadiene. The microstructure of the polymers was determined by ^1^H NMR, ^13^C NMR, and IR spectra. The microstructure of polymers was determined as reported in the literature [69].

For the method of IR, ratios of *cis*-1,4, 1,2, and *trans*-1,4 structures for the polybutadiene were determined from the absorption bands at 738, 911, and 967 cm^−1^.

*cis*-1,4 content (%) = (17,700 × A_738_/B) × 100

1,2 content (%) = (3670 × A_911_/B) × 100

*trans*-1,4 content (%) = (4740 × A_967_/B) × 100

where

B = 17,700 × A_738_ + 3670 × A_911_ + 4740 × A_967_

A_738_, A_911_, and A_967_ are the absorbances at 738, 911, and 967 cm^−1^, respectively.

For the method of ^1^H NMR, ratios of *cis*-1,4, 1,2- and *trans*-1,4 structures for the polybutadiene were determined from the resonance peaks at 5.38, 4.98, and 5.42 ppm.

$$1,2\;\mathrm{content}\;\%\;\;\frac{I_{h}}{I_{g}+I_{a}+I_{d}+\frac{I_{h}}{2}}$$

cis-1,4/trans-1,4=IdIa

*cis*-1,4 + *trans*-1,4 (%) = 100% − 1,2 content (%),

from which the *cis*-1,4 and *trans*-1,4 content can be calculated. In these equations, *I*_a_, *I*_d_, *I*_g_, *I*_h_ are integrated areas for peaks a, d, g, and h.

### 2.3. Procedure for Nickel-Catalyzed Butadiene Polymerization

All the manipulations were performed under an atmosphere of dry nitrogen. The hexane solution of 1,3-butadiene, toluene solution of Ni(acac)_2_, and alkylaluminium were sequentially injected into the reactor, and the polymerization was left to be stirred for 4 h at the set temperature. The polymerization was quenched by excessive ethanol. The polymer was washed repeatedly with ethanol and dried under vacuum at 50 °C to constant weight. The polymer yield was determined by gravimetry.

## 3. Results

**Figure 1:**
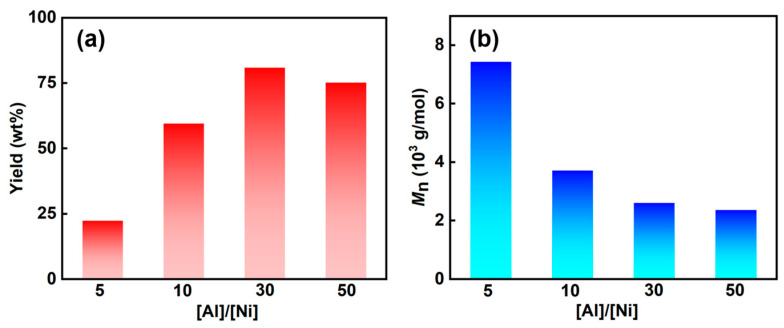
Effects of different [Al/Ni] ratios on yield and *M*_n_. (**a**) yield; (**b**) *M*_n_.

The polymerization studies commenced by evaluating the amount of cocatalyst on the overall polymerization performance. As summarized in Table 1, entries 1–4, the Al/Ni ratio has a pronounced influence on the catalytic activity and molecular weights, as well as the microstructure of the obtained LPB products. As the Al/Ni ratio increased from 5 to 50, the yields of LPB first increased gradually from 80.9% and then slightly decreased to 75.1% (Figure 1a). The reason for the low yield at Al/Ni = 5 is ascribed to its insufficient initiation; beyond this value, more nickel precatalyst can be turned into effective active species and thus results in gradually increased yields from 59.1% to 80.9%. Under a high Al/Ni ratio of 50, a slightly lower LPB yield is again witnessed, probably due to the overreduction of nickel active species under such high cocatalyst concentration. These studies also revealed an optimal Al/Ni ratio of 50, which will be adopted in the following reactions. Regarding the molecular weight of the obtained LPBs, their *M*_n_s decreased gradually from 7.43 × 10^3^ g/mol to 2.36 × 10^3^ g/mol upon increasing the Al/Ni ratio from 5 to 50 (Figure 1b). Such a result indicates that the formed active species is very sensitive to this cocatalyst, and higher cocatalyst concentrations promote more frequent chain transfer events with the alkylaluminum species. Such a presumption of chain transfer can also be inferred from broad molecular weight distribution under a high Al/Ni ratio of 50, under which a PDI of 3.63 is demonstrated, which is much larger than those obtained from Al/Ni ratios of 10 and 30, respectively. Regarding the microstructure of the obtained LPBs, varying Al/Ni ratios imposed very little influence on the total 1,4 content, giving values in the range of 98.8–99.3%, and vinyl content staying below ca. 1%. Nevertheless, *cis*-1,4 content seems to be affected by the Al/Ni ratio. Upon increasing the Al/Ni ratio from 10 to 50, *cis*-1,4 content decreased from 79.8% to 75.7%, and simultaneously *trans*-1,4 content increased from 18.9% to 23.5%.

**Figure 2:**
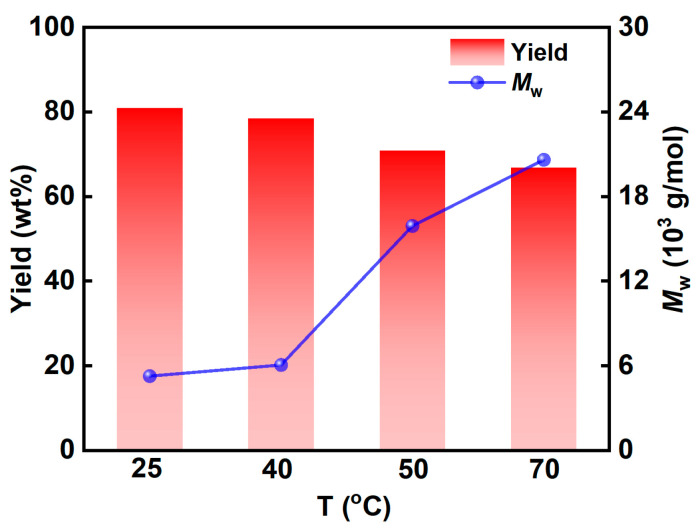
Effects of different polymerization temperatures on yield and *M*_W_.

**Figure 3:**
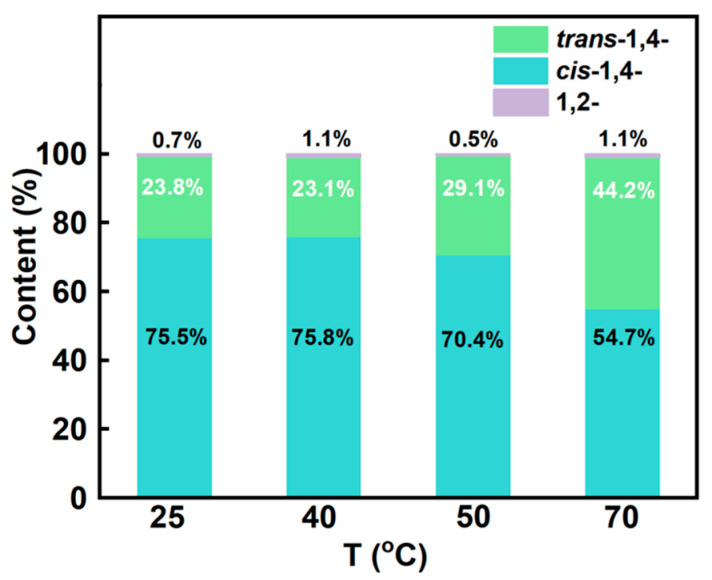
Effects of different polymerization temperatures on microstructures.

Polymerization temperatures were next examined and found to significantly impact catalytic efficiency and stereo-controlling. Under relatively low temperature conditions (25–40 °C), much higher yields of 80.9% and 78.5% were achieved, respectively, demonstrating good catalyst robustness. However, increasing the temperature beyond this point led to a pronounced decline in activity, with yields falling to 70.9% at 50 °C and 66.8% at 70 °C (Figure 2). Such thermal sensitivity is well documented for late-transition metal catalysts and is generally attributed to decomposition of the active propagating species. In terms of molecular weight, varying temperature seemed to bring subtle influence on the number-average molecular weight *M*_n_, but the weight-average molecular weight varied significantly. As the blue curves show in Figure 2, obviously increased *M*_w_ values were observed. These results indicate facilitated chain transfer reactions under high temperatures. Such a presumption can also be concluded from the broad molecular weight distributions under high temperatures. The PDI broadened from 2.02 to 7.56 when the temperature increased from 25 °C to 70 °C. The observed broadening of polydispersity may also be attributed to partial decomposition of the active nickel species at elevated temperatures, generating multiple catalytic sites, as late-transition metal catalysts are generally known to exhibit limited thermal stability in olefin polymerization [40,70]. Temperature also strongly affects regio- and stereoselectivity. *Cis*-1,4 content gradually decreases, while *trans*-1,4 steadily increases with temperature (Figure 3), indicating a shift from kinetically controlled *cis*-selective propagation at lower temperatures to a more thermodynamically governed insertion manner. This trend aligns well with prior mechanistic studies, which propose that higher temperatures facilitate interconversion of *anti*-*η*^3^-allyl species (yielding *cis*-1,4 units) into *syn*-*η*^3^-allyl configurations (responsible for *trans*-1,4 incorporation) [71]. At 70 °C, *cis*-1,4 content drops sharply to 54.7%, as shown in Figure 3, marking a threshold beyond which stereo-controlling is severely compromised. Despite these variations, the total 1,4 content remains high across the examined temperature range, and vinyl incorporation stays negligible under all conditions tested, with values of 0.5~1.1% (Figure 3). This result shows a clear contrast to *n*-BuLi-mediated anionic polymerization, where high 1,4 content is very difficult to achieve. Considering the broadened molecular weight distributions and reduced cis-1,4 content at elevated temperatures, polymerization should preferably be conducted below 40 °C to obtain LPB with improved low-temperature performance.

Polymerization solvent further affects the catalytic outcome. Comparing experiments conducted on toluene and cyclohexane under otherwise identical conditions reveals distinct trends in activity, molecular weight, and microstructure (Table 1, entries 8–9). Toluene yields a conversion of 84.0%, slightly outperforming cyclohexane (79.2%) and *n*-hexane (80.9%). Additionally, polymers obtained in toluene exhibit appreciably higher molecular weights (*M*_n_ = 5.46 × 10^3^ g/mol vs. 2.83 × 10^3^ g/mol in cyclohexane), implying that chain transfer processes are suppressed in aromatic media. Microstructural analysis further highlights solvent effects: polymerization in toluene affords the highest *cis*-1,4 selectivity (~86%), whereas cyclohexane yields a more typical *cis/trans* ratio (~76/22). Interestingly, vinyl units rise sharply in toluene (~6.6%), suggesting partial loss of control in the aromatic medium. These combined results underscore that solvent polarity modulates both the kinetic balance between propagation and chain transfer and the stereochemical outcome of insertion, offering a straightforward means to fine-tune polymer properties in this system.

**Figure 4:**
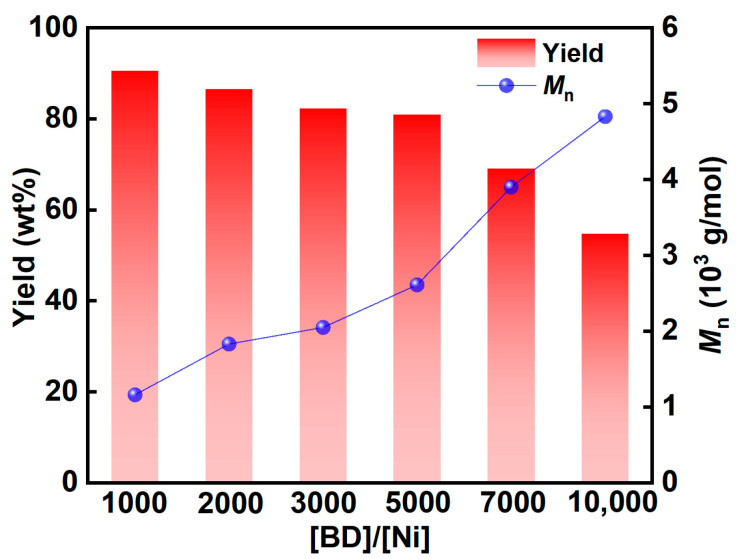
Effects of different [Bd]/[Ni] ratios on yield and *M*_n_.

Molecular weight plays a determining role in governing the properties of liquid rubber. In addition to the above-mentioned conditions, such as Al/Ni ratio and polymerization temperatures, the molar ratio between monomer and catalysts also serves as an effective regulator of molecular weights. Based on this consideration, Ni-mediated polymerization was further carried out under different [Bd]/[Ni] ratios. As shown in Table 2, as the [Bd]/[Ni] ratio increases, a clear trend in molecular weight evolution is observed. Specifically, *M*_n_ increased progressively from 1.16 × 10^3^ g/mol to 4.83 × 10^3^ g/mol with increasing the [Bd]/[Ni] ratios from 1000 to 10,000 (Figure 4), indicating that higher monomer availability relative to active Ni centers favors longer chain growth prior to chain transfer or termination events. From the blue curve shown in Figure 4, gradually increasing *M*_n_ values can be observed. This behavior is consistent with a coordination/insertion mechanism in which chain propagation competes with aluminum-mediated chain transfer; increasing the monomer concentration effectively enhances the propagation rate relative to chain transfer processes, thereby affording higher molecular weight products. Nevertheless, it was found that the *M*_n_s are not increased linearly with the increased [Bd]/[Ni] ratios, which indicates the polymerization deviates from living polymerizations. This behavior makes sense when considering the common chain transfer behavior to butadiene monomer for Ni-mediated polymerization systems. Similar results have been observed in previous reports [68,72,73].

In contrast, the polymer yield shows an inverse dependence on [Bd]/[Ni]. At relatively low monomer-to-catalyst ratios, high yields are typically achieved due to sufficient catalyst concentration and efficient monomer consumption within the fixed reaction time. However, as [Bd]/[Ni] increases, the yield decreases slightly. For instance, when the [Bd]/[Ni] ratio reaches 10,000, a yield of only 54.7% was demonstrated. This result can be attributed to incomplete monomer conversion under reduced effective catalyst concentration per monomer unit. In other words, although higher [Bd]/[Ni] promotes chain extension, it may simultaneously limit overall conversion efficiency when the number of active sites becomes insufficient to fully polymerize the increased monomer feed.

**Figure 5:**
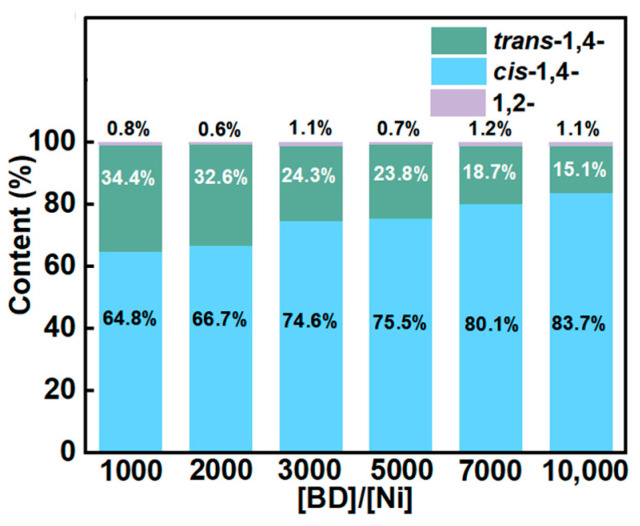
Effects of different [Bd]/[Ni] ratios on microstructures.

The stereochemical outcome is also influenced by the [Bd]/[Ni] ratio. As shown in Figure 5, with increasing [Bd]/[Ni], the *cis*-1,4 content exhibits a gradual increase from 64.8% to 84.7%, with a simultaneous gradual decrease in *trans*-1,4 content from 34.1% to 15.1%. The 1,2 content was negligibly influenced, again indicating the high 1,4 selectivity of the system, regardless of [Bd]/[Ni] ratios. The detailed mechanism is still unclear, but it may be related to the steric congestion around the metal center. A higher molecular weight polymer caused increased steric congestion around the metal center, forcing the butadiene to coordinate the nickel center in a *cis*-1,4-*η*^4^-, which is the key structure to generate *cis*-1,4 units.

**Figure 6:**
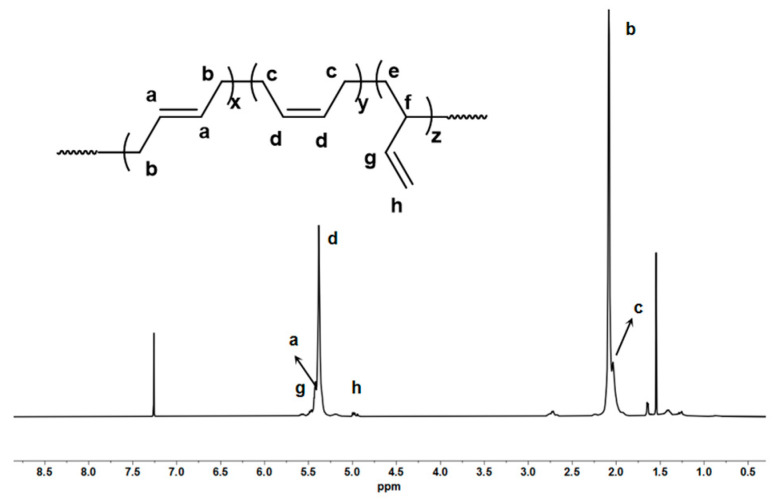
^1^H NMR of the LPB (Table 1, entry 3).

**Figure 7:**
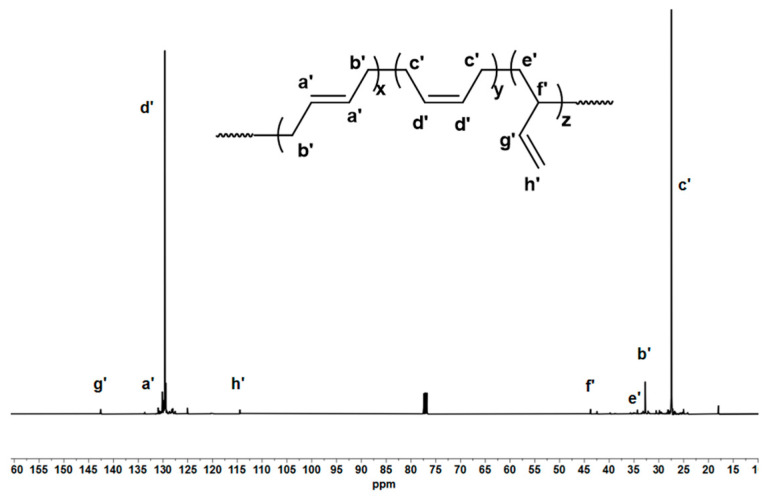
^13^C NMR of the LPB (Table 1, entry 3).

A representative LPB sample from Table 1 entry 3 was characterized by ^1^H and ^13^C NMR spectroscopy to elucidate its microstructure. As shown in Figure 6 and Figure 7, the methine protons in *cis-*1,4 and *trans*-1,4 units (H_d_, H_c_) appear at 5.38 and 5.42 ppm, respectively, while the methylene protons adjacent to the C=C double were found at 2.08 and 2.04 ppm. The proton =CH_2_ in the vinyl unit is located at 4.98 ppm. In the ^13^C NMR spectrum, the characteristic carbon signals (C_d’_ and C_c’_) in the *cis-*1,4 units are found at 129.58 and 27.45 ppm, respectively, whereas those of the *trans-*1,4 units (C_a’_ and C_b’_) were found at 130.09 and 32.74 ppm. Additionally, the vinyl carbons C_h’_ and C_g’_ were found at 142.51 and 114.16 ppm. From these assignments, the cis-1,4 content was calculated as 76.2%, the trans-1,4 content was calculated as 23.0%, and the 1,2 content was calculated as 0.8%, which are very close to the values calculated from IR.

In late-transition-metal-mediated diene polymerization, regioselectivity is generally strongly influenced by external donors, with donor incorporation generally shifting the 1,4 selectivity toward 1,2 [42,74]. Based on this, the influence of various external donors on the Ni(acac)_2_/EASC catalytic system was next investigated, and the results are summarized in Table 3. It was found that the introduction of different donors exerts markedly distinct effects on catalytic activity, molecular weight regulation, and microstructure. Notably, donors bearing strongly coordinating oxygen- or sulfur-containing functionalities, such as 1,4-cyclohexanedione (CHD), 2-aminothiophenol (ATP), and pentafluorophenol (PFP), lead to a slight decrease in polymer yield compared with the donor-free system. The yields drop from 80.9% to approximately 67–69%, indicating partial deactivation of the catalytic center. This behavior can be reasonably attributed to strong coordination of the O and S heteroatoms to the nickel center, which competes with butadiene coordination and reduces the concentration of active propagation species. Such strong binding may also alter the electronic structure of the Ni center, further suppressing catalytic turnover. In contrast, N- and P-containing donors, such as azobisisobutyronitrile (AIBN), azobis(2,4-dimethylvaleronitrile) (AVBN), diethyl phosphite (DEP), and triphenyl phosphate (TPP), do not cause obvious catalytic deactivation, leading to comparable or even slightly higher values than the donor-free system. These results demonstrate the relatively good tolerance of the generated Ni-based active species toward N- and P-containing donors, showing contrast to O and S heteroatoms.

Incorporation of external donors also had a large influence on the molecular weight and molecular weight distributions of the products. Compared to the donor-free system, upon addition of O and S donors, although the number-average molecular weight (*M*_n_) of the obtained polybutadienes did not change a lot, their weight-average molecular (*M*_w_) increased significantly. For instance, *M*_w_s increased to 6.65 × 10^3^ g/mol for CHD and 7.81 × 10^3^ g/mol for ATP, whereas their *M*_n_s witnessed little change. In contrast, for N donors, both the *M*_n_s and *M*_w_s increased significantly, as evidenced by the increments of *M*_n_s to 3.25 × 10^3^ g/mol for AIBN and 3.85 × 10^3^ g/mol for AVBN and increments of *M*_w_s to 9.82 × 10^3^ g/mol for AIBN and 13.17 × 10^3^ g/mol for AVBN. The general increase in molecular weight upon donor addition can be rationalized by coordination of the donor to the active nickel center, which increases steric congestion around the metal site. This steric shielding likely suppresses chain transfer events, whether to aluminum species or to monomer, thereby prolonging chain propagation and leading to higher molecular weights. Furthermore, because of the coordination of external donors to the active species, which also may cause an inconsistently structured multi-site active species, polymers with obviously broader molecular weight distributions were also witnessed. Attempts to regulate the polydispersity by varying the concentration of donors proved to be ineffective, as evidenced from the results shown in entries 8–11, which show that changing the TPP/Ni ratio from 1 to 10 caused little improvement in the molecular weight distributions. Nevertheless, it was found that at higher TPP concentrations (D/Ni = 10), a significant decrease in LPB yield is observed, which is likely attributable to overcoordination of TPP to the nickel active species, thereby reducing the availability of vacant coordination sites required for monomer coordination and insertion.

**Figure 8:**
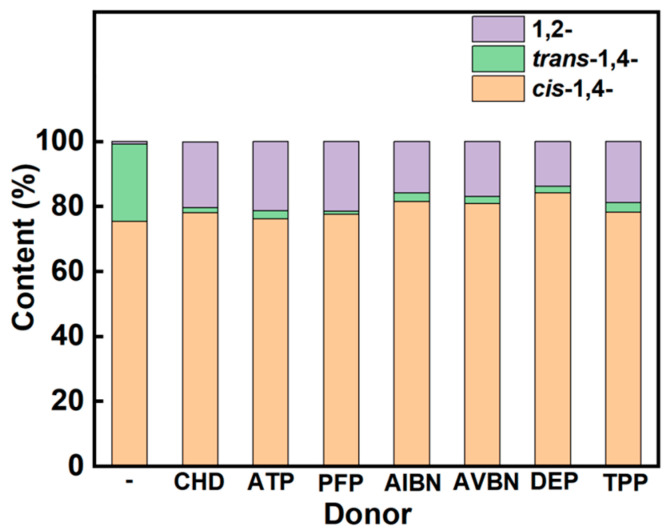
Effects of different external donors on microstructures.

**Scheme 2:**
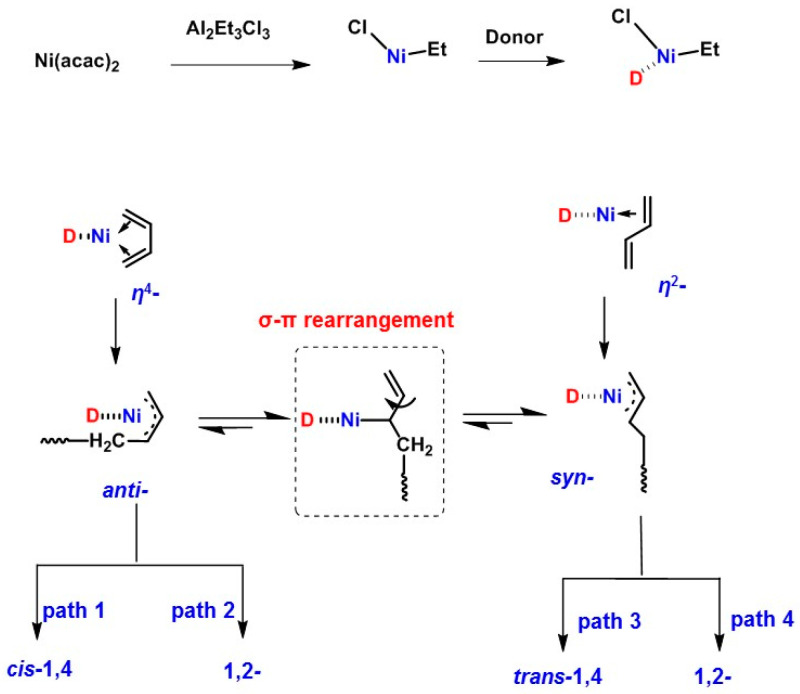
Plausible mechanism for butadiene polymerization.

One of the most prominent changes induced by donor addition is observed in the polymer microstructure. As shown in Table 3 and Figure 8, compared with the donor-free system, the incorporation of external donors leads to a substantial decrease in the total 1,4 content and a dramatic increase in 1,2-vinyl incorporation, from 0.7% up to 15–21%. Additionally, it was found that the content of *cis*-1,4 content did not change significantly, with only the *trans*-1,4 content decreasing. For instance, in systems involving CHD, ATP, and PFP, the *cis*-1,4 content remained comparable to that of the donor-free counterpart, yielding values of 78.1%, 76.3%, and 77.6%, respectively. In contrast, the *trans*-1,4 content decreased from 23.8% to only 1.0~2.4%, with the corresponding conversion to 1,2 content of 20.2–21.4%. This striking shift indicates that donor coordination strongly perturbs the monomer insertion mode. As illustrated in the plausible scheme (Scheme 2), for the donor-coordinated nickel active species, the butadiene monomer can coordinate via three modes: *cis*-1,4-*η*^4^, *trans*-1,4-*η*^4^, and 1,2-*η*^2^. The *cis*-1,4-*η*^4^-coordinated intermediates form the *anti*-π-allyl-*η*^3^ active species, which serves as the main intermediate for the generation of *cis*-1,4- and 1,2-polybutadienes. In contrast, the *syn*-π-allyl-*η*^3^ active species is formed either from *trans*-1,4-*η*^4^ and 1,2-*η*^2^ coordination modes or via σ-π rearrangement of the *anti*-π-allyl-*η*^3^ structure, ultimately producing trans-1,4- and 1,2-polybutadienes. The formation of the former active species (*anti*-π-allyl-*η*^4^) is generally kinetically controlled. In donor-involved reactions, it was found that the polymerization rate was only slightly influenced by the donors; therefore, it is presumed that donor incorporation exerts only a minor effect on the formation of the *anti*-π-allyl-*η*^3^ active species. In contrast, the formation of the *syn*-π-allyl-*η*^3^ structure is often not kinetically controlled. Due to the donor molecules occupying vacant coordination sites on the nickel center, the butadiene monomer prefers to coordinate to the active species in a 1,2-*η*^2^ mode, thereby forming the vinyl structure via path 4.

**Figure 9:**
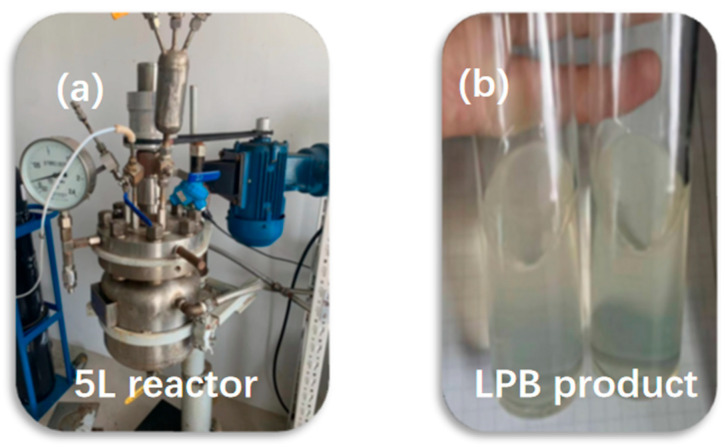
5 L scale-up experiment for LPB. (**a**) 5 L scale-up device; (**b**) LPB product.

Given the simplicity of the Ni(acac)_2_/EASC catalytic system, along with its high activity and excellent *cis*-1,4 selectivity in hexane, two scale-up experiments at different [Bd]/[Ni] ratios (10,000 and 7000) were performed in a 5 L polymerization reactor. The results are summarized in Figure 9. Under conditions of Al/Ni = 20 at 25 °C, LPB products were obtained in yields of 73.8% and 92.6%, respectively. As shown in Figure 9b, both products exhibit typical liquid states, with *M*_n_s of 3.8 × 10^3^ and 2.9 × 10^3^ g mol^−1^, respectively, and relatively narrow molecular weight distributions (2.31 and 2.25), consistent with the trends observed in Table 2. Detailed microstructural analysis reveals high 1,4 content of 98.9% and 98.5%, respectively. For [Bd]/[Ni] = 10,000, the polymer consists of 83.9% *cis*-1,4 and 15.0% *trans*-1,4 units, whereas for [Bd]/[Ni] = 7000, 81.0% *cis-*1,4 and 16.2% *trans*-1,4 units are obtained.

## 4. Conclusions

In summary, a ligand-free Ni(acac)_2_/EASC catalytic system has been established as an efficient and practical approach for the synthesis of low molecular weight liquid polybutadiene with predominantly 1,4 microstructure. Systematic evaluation of reaction parameters demonstrates that the catalytic performance and polymer properties can be finely tuned over a broad range.

The Al/Ni ratio plays a decisive role in regulating catalytic activity and chain transfer behavior. Increasing the Al/Ni ratio from 5 to 50 enhances the yield from 22.3% to ~80%, while simultaneously decreasing the number-average molecular weight (*M*_n_) from 7.43 × 10^3^ to 2.36 × 10^3^ g mol^−1^ and broadening the dispersity (PDI up to 3.63), indicating facilitated chain transfer processes at high cocatalyst loadings. In contrast, the overall 1,4 content remains consistently high (98.8–99.3%), although a slight shift from *cis*-1,4 to trans-1,4 units is observed. Polymerization temperature significantly influences both catalytic stability and stereoselectivity. While high yields (~80%) are maintained at 25–40 °C, further increasing the temperature to 70 °C results in decreased activity (66.8%) and substantially broader molecular weight distributions (PDI up to 7.56). More importantly, the *cis-*1,4 content decreases markedly from ~75% to 54.7%, accompanied by an increase in *trans-*1,4 content to 44.2%, highlighting a temperature-induced shift toward thermodynamically favored insertion pathways. The [Bd]/[Ni] ratio provides an effective handle for molecular weight control. Increasing this ratio from 1000 to 10,000 leads to a steady increase in *M*_n_ from 1.16 × 10^3^ to 4.83 × 10^3^ g mol^−1^, while the polymer yield decreases from 90.5% to 54.7%, reflecting reduced catalyst availability per monomer unit. Concurrently, the *cis*-1,4 content increases from 64.8% to 83.7%, with a corresponding decrease in *trans*-1,4 content, suggesting that higher monomer concentrations favor *cis*-selective insertion.

External donors exert the most pronounced influence on regioselectivity. Strongly coordinating O- and S-containing donors slightly reduces catalytic activity and dramatically increases 1,2-vinyl content from 0.7% to 20.2–21.4%, primarily at the expense of *trans*-1,4 units, while largely preserving cis-1,4 selectivity (~76–78%). In contrast, N- and P-containing donors (e.g., AIBN, AVBN, DEP, TPP) show minimal deactivation and can increase *M*_n_ up to 3.85 × 10^3^ g mol^−1^, albeit with broader dispersities. These findings confirm that donor coordination modulates the insertion mode and alters the balance between competing propagation pathways.

Overall, this work demonstrates that a simple nickel-based system can deliver LPB with tunable molecular weight (1.16–7.43 × 10^3^ g mol^−1^), high 1,4 content (up to 99.3%), and adjustable microstructure through rational control of reaction parameters. The combination of mechanistic insight, operational simplicity, and scalability highlights the potential of this system as a cost-effective alternative for the production of liquid polybutadiene and related materials.

## Figures and Tables

**Table 1:** Butadiene polymerization by Ni(acac)_2_/EASC under different conditions ^a^.

Run	[Al]/[Ni]	T (°C)	Yield (wt%)	*M*_n_ ^b^ (×10^3^)	*M*_w_ ^b^ (×10^3^)	PDI ^b^	*cis-*1,4 ^c^	*trans*-1,4 ^c^	1,2 ^c^
1	5	25	22.3	7.43	31.10	4.18	-	-	-
2	10	25	59.5	3.71	8.35	2.28	79.8	18.9	1.2
3	30	25	80.9	2.61	5.27	2.02	75.5	23.8	0.7
4	50	25	75.1	2.36	8.57	3.63	75.7	23.5	0.7
5	30	40	78.5	2.37	6.06	2.56	75.8	23.1	1.1
6	30	50	70.9	2.71	15.9	5.89	70.4	29.1	0.5
7	30	70	66.8	2.72	20.6	7.56	54.7	44.2	1.1
8 ^d^	30	25	84.0	5.46	10.8	1.99	86.2	7.1	6.6
9 ^d^	30	25	79.2	2.83	7.19	2.54	76.3	22.4	1.3

^a^ Polymerization conditions: [Bd] = 1.85 M, [Bd]/[Ni] = 5000, 20 mL, *n*-hexane, 4 h. ^b^ *M*_n_, *M*_w,_ and PDI were determined by GPC using polystyrene standards. ^c^ Determined by IR and NMR. ^d^ Toluene and cyclohexane as solvents.

**Table 2:** Butadiene polymerization by Ni(acac)_2_/EASC under different [Bd]/[Ni] ratios ^a^.

Run	[Bd]/[Ni]	Yield (wt%)	*M*_n_ ^b^ (×10^3^)	*M*_w_ ^b^ (×10^3^)	PDI ^b^	*cis-*1,4 ^c^	*trans*-1,4 ^c^	1,2 ^c^
1	1000	90.5	1.16	2.44	2.10	64.8	34.4	0.8
2	2000	86.5	1.83	4.19	2.28	66.7	32.6	0.6
3	3000	82.3	2.05	5.29	2.62	74.6	24.3	1.1
4	5000	80.9	2.61	5.27	2.02	75.5	23.8	0.7
5	7000	69.1	3.90	7.30	2.52	80.1	18.7	1.2
6	10,000	54.7	4.83	10.7	2.21	83.7	15.1	1.1

^a^ Polymerization conditions: [Bd] = 1.85 M, [Al]/[Ni] = 30, 20 mL, *n*-hexane, 4 h. ^b^ *M*_n_, *M*_w_, and PDI were determined by GPC using polystyrene standards. ^c^ Determined by IR and NMR.

**Table 3:** Butadiene polymerization by Ni(acac)_2_/EASC in the presence of different external donors ^a^.

Run	Donor	Yield (wt%)	*M*_n_ ^b^ (×10^3^)	*M*_w_ ^b^ (×10^3^)	PDI ^b^	*cis-*1,4 ^c^	*trans*-1,4 ^c^	1,2 ^c^
1	-	80.9	2.61	5.27	2.02	75.5	23.8	0.7
2	CHD	68.2	2.13	6.65	3.12	78.1	1.6	20.2
3	ATP	69.0	2.38	7.81	3.28	76.3	2.4	21.3
4	PFP	67.4	2.85	6.90	2.42	77.6	1.0	21.4
5	AIBN	83.8	3.25	9.82	3.02	81.6	2.6	15.9
6	AVBN	82.9	3.85	13.17	3.42	80.9	2.2	17.0
7	DEP	76.3	3.68	14.80	4.02	84.2	2.1	13.7
8	TPP	83.8	1.43	5.02	3.51	78.2	3.1	18.7
9 ^d^	TPP	84.5	1.56	5.19	3.33	78.1	3.0	18.9
10 ^d^	TPP	86.2	1.55	5.41	3.49	78.6	4.3	17.1
11 ^d^	TPP	48.9	1.48	6.21	4.20	76.9	3.8	19.3

^a^ Polymerization conditions: [Bd] = 1.85 M, [Al]/[Ni] = 30, [D]/[Ni] = 3, 20 mL, *n*-hexane, 4 h. ^b^ *M*_n_, *M*_w_, and PDI were determined by GPC using polystyrene standards. ^c^ Determined by IR and NMR. ^d^ [D]/[Ni] = 1, 5 and 10 were applied, respectively.

## Data Availability

The original contributions presented in this study are included in the article. Further inquiries can be directed to the corresponding author.
